# Long-Term Donor Site Morbidity and Flap Perfusion Following Radial versus Ulnar Forearm Free Flap—A Randomized Controlled Prospective Clinical Trial

**DOI:** 10.3390/jcm11133601

**Published:** 2022-06-22

**Authors:** Daniel G. E. Thiem, Fabia Siegberg, Paul Römer, Sebastian Blatt, Andreas Pabst, Diana Heimes, Bilal Al-Nawas, Peer W. Kämmerer

**Affiliations:** 1 Department of Oral and Maxillofacial Surgery, University Medical Centre Mainz, 55131 Mainz, Germany; fabia.siegberg@icloud.com (F.S.); paul.roemer@unimedizin-mainz.de (P.R.); sebastian.blatt@unimedizin-mainz.de (S.B.); diana.heimes@unimedizin-mainz.de (D.H.); bilal.al-nawas@unimedizin-mainz.de (B.A.-N.); peer.kaemmerer@unimedizin-mainz.de (P.W.K.); 2 Department of Oral and Maxillofacial Surgery, Federal Armed Forces Hospital, Rübenacherstr. 170, 56072 Koblenz, Germany; andreas.pabst@uni-mainz.de

**Keywords:** RFFF, UFFF, reconstruction, head and neck, hyperspectral imaging, HSI, randomized controlled prospective study, free flap, donor site morbidity

## Abstract

This clinical prospective randomized controlled study aimed to investigate the differences between Radial (RFFF) and Ulnar (UFFF) Forearm Free Flap in terms of success, performance, and donor site morbidity. Thirty patients with reconstruction of the head and neck region were included. For the first time, this study assessed flap-perfusion characteristics, donor-site-wound-healing dynamics and hand perfusion using hyperspectral imaging. Further, subjective (Likert-scale, DASH-score) and objective (grip/pinch-strength) parameters of donor site morbidity were analysed. Postoperative follow-up was performed until 6 months after index surgery. With 100% of patients, RFFF and UFFF were equally successful. Compared to surrounding reference, UFFF revealed significant lower tissue oxygenation saturation (StO_2_) than RFFF. Compared with UFFF, blood flow in both the thenar and hypothenar region were significantly reduced 6 months following RFFF transfer. After four weeks, 27% more patients demonstrated impaired wound healing following RFFF transfer. After 6 months, epithelial-surface continuity was restored in all patients of both groups. After 6 months, overall rates of both subjective and objective donor site morbidity were comparable between RFFF and UFFF. RFFF and UFFF both demonstrate similar success rates and HSI-perfusion dynamics following transfer. After 4 weeks, wound-healing disorder appeared significantly more often in RFFF than in UFFF; however, they became equal after 6 months. RFFF and UFFF can be considered as mutual alternatives.

## 1. Introduction

In reconstructive oral and maxillofacial surgery, microsurgical flap transfer is one of the most important and regularly performed methods for defect reconstruction of the head and neck region. Flap survival as the primary criterion for success after microvascular flap transfer is generally considered to be very good at >94% [1]. However, donor site morbidity cannot be neglected in this context. Complications range from impaired wound healing to significant scarring and functional limitations such as impaired sensitivity and/or mobility. Among the many different types of flaps with various overlapping indications, the inner side of the forearm is one of the most common donor sites for microvascular reconstruction of soft tissue defects, not only in the head and neck region, but also for the entire body. Major advantages of the radial and ulnar forearm flap include their straightforward harvesting technique with a generally uniform anatomy with few anatomical variations and typically adequate length of the vascular pedicle (>15 cm) [2]. While the radial forearm free flap (RFFF), first described by Soutar et al. in 1983, was considered a simple and versatile flap, the use of the ulnar forearm free flap (UFFF), first described by Lovie et al. in 1984, became less popular. This was due to the incorrect assumption that the UFFF artery is the dominant vascular supply to the palm [3,4]. The elastic and soft nature of the skin is particularly suitable for reconstructions of the head and neck region and frequently provides good functionality and aesthetics [5,6]. The above-mentioned criteria lead to different indications with regard to the choice between UFFF and RFFF. Harvesting of both flaps can only be performed if sufficient blood supply to the hand via the palmar arches has been ensured beforehand. More than 15 s are considered critical, indicating impaired blood circulation with the increased risk for palmar malperfusion [7]. According to the literature, flap survival can be considered equal between RFFF and UFFF, with the radial vascular pedicle being usually 3–4 cm longer and slightly larger in caliber [8]. In contrast, there is a risk of damage to the terminally branching superficial radial nerve in up to 30% of patients with partial or complete temporary or permanent loss of sensory innervation of the radial-lateral skin of the thumb and the dorsal side of the 2nd and 3rd fingers [5], as well as reduced skin hairiness in the ulnar (medial) inner forearm area [9]. However, fewer wound healing disorders and better aesthetic outcomes following the donor sites’ full-thickness skin coverage were found on the ulnar since the surface consists of well-perfused muscle instead of the tendinous surface of the radial site [10]. Established techniques to improve healing include full-thickness skin graft incision to ensure subdermal fluid drainage, the additional use of vacuum therapy, or the use of overknot bandages to provide direct contact of the skin graft with the wound bed [11]. Superiority of one of the methods has not been demonstrated, which is consistent with a recent meta-analysis [11,12]. The primary objective of the present study was to quantify wound healing at the donor site using hyperspectral imaging, a biophotonic sensor technique for the investigation of biochemical tissue properties (details given under 2.2) and to assess overall donor site morbidity in a comparison between RFFF and UFFF. The secondary objective was to characterise flap perfusion in a comparison of RFFF and UFFF. We hypothesise that RFFF is superior to UFFF regarding donor site morbidity and overall flap performance.

## 2. Materials and Methods

### 2.1. Patients

This study was approved by the local ethics committee of Rhineland-Palate (registration number: 2020-15436) and was conducted in accordance with the protocol and in compliance with the moral, ethical, and scientific principles governing clinical research as set out in the Declaration of Helsinki of 1975 and revised in 1983. Inclusion criteria were the need for defect reconstruction using RFFF or UFFF, clinical equivalence when comparing RFFF and UFFF (skin surface without scars, no previous surgery in the area of the harvest region), a preoperative unremarkable Allen test with a re-capillarization time following compression of ulnar = radial artery (<15 s), and the patient’s ability to consent to study participation. Patients who did not meet the inclusion criteria, patients < 18 years of age, cases without regular hyperspectral imaging as specified below, and those with manifest peripheral neuropathy (diabetic neuropathy or leprosy) were excluded. Informed consent was obtained from all patients prior to participation in the study. Randomization was performed computationally by using the “Trial Randomization Tool” (National Cancer Intitute; https://ctrandomization.cancer.gov/tool/; accessed on 3 March 2020). However, randomization was performed only on the nondominant side, or in the case of a negative Allen test, whereas decision was independent on the defects’ type, site, or size. All surgical procedures were performed by two different experienced surgeons at the department of oral and maxillofacial surgery, facial plastic surgery, University Medical Centre Mainz, Johannes Guttenberg University Mainz, Mainz, Germany in the period from October 2020 until December 2021. Allen’s test (the only mandatory test) and Hyperspectral Imaging were routinely performed before surgery to demonstrate adequate blood flow from the radial or ulnar artery to the hand [7].

### 2.2. Monitoring

I. Hyperspectral perfusion monitoring of:
  (a) RFFF and UFFF: The measurement time points of hyperspectral perfusion monitoring were preoperatively/baseline (t0), after flap preparation (t1), after arterial and venous anastomosis (t2), after flap insertion (t3), and at regular intervals until 72 h (t4–t10) after flap insertion. The parameters included were tissue oxygen saturation (StO_2_), near infrared perfusion index (NPI), tissue hemoglobin index (THI) as a surrogate parameter of microvascular blood flow, as well as tissue water index (TWI). Detailed parameter information was described previously [13].
  (b) Thenar and hypothenar eminence: The measurement time points of thenar and hypothenar perfusion monitoring using hyperspectral imaging (HSI) were baseline (prior flap raise) (t0), 10 days after surgery (t1), 4 weeks after surgery (t2), and 6 months after surgery (t3).
II. Quantification of donor site wound healing dynamics via Hyperspectral Imaging

**Figure 1:**
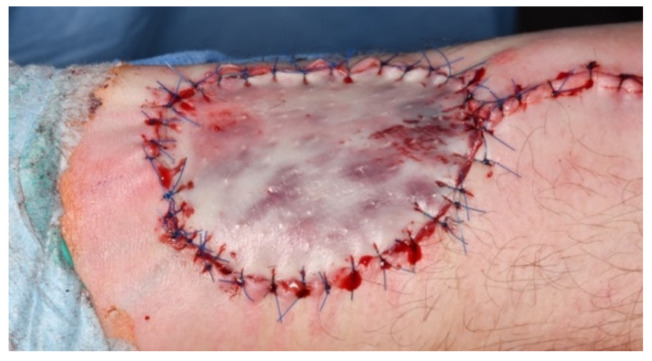
Clinical situation after closure of the RFFF donor site using full-thickness skin graft from the groin.

**Figure 2:**
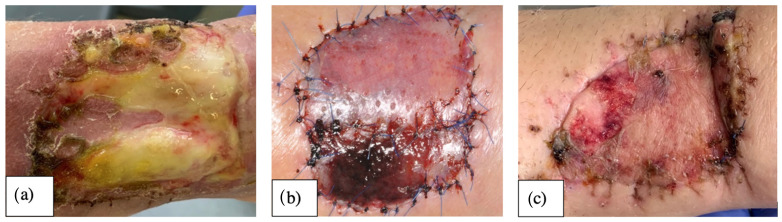
Shows the extent of impaired wound healing after coverage with full-thickness skin graft from the groin at different time points and with different success (**a**) complete loss, (**b**) incomplete integration and (**c**) complete integration).

In all cases, the donor site was covered using a full-thickness skin graft from the groin (Figure 1). In order to prevent subdermal seroma formation, we performed skin graft incisions and overknot bandage coverage. The baseline situation was assessed prior to flap preparation (t0). We evaluated the success of full-thickness healing as a result of primary wound healing on the 10th postoperative day during removal of the overknot bandage using hyperspectral imaging (t1). Depending on the epithelialized donor site, healing was classified into (a) complete loss of the full-thickness skin graft (100% of the donor site [cm^2^]), (b) loss of 99% to 75%, (c) loss of 74.9% to 50%, (d) loss of 49.9% to 25%, (e) 24.9% to 1%, and (f) complete integration (100% of the donor site in cm^2^) (Figure 2). Further hyperspectral quantification of wound healing was performed on postoperative day 28 (4 weeks) (t2), and 6 months after surgery (t3). Areas of the donor site that did not show healing on postoperative day 10 were assigned to secondary wound healing in the course of follow-up and were thus not included in the subsequent HSI quantification at t2 and t3.

III. Hyperspectral Imaging (HSI)

**Figure 3:**
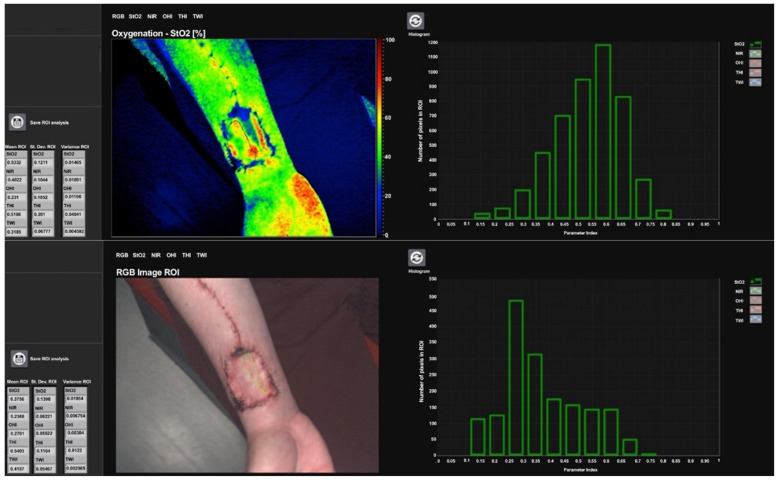
Shows full-thickness skin coverage after RFFF on postoperative day 10 with manually drawn region of interest in projection over the underlying flexor ligament muscle bellies (**top**) with an oxygen saturation of 53.3%, and the drawn region of interest in projection over the flexor carpi radialis muscle tendon (**bottom**) with a tissue oxygen saturation (StO_2_) of 37.6%.

Hyperspectral quantification was conducted using a hyperspectral sensor system (TIVITA^TM^ Tissue System, Diaspective Vision GmbH, Pepelow, Germany) with the camera-specific software package (TIVITA^TM^ Suite) in accordance with the literature [14]. The parameters used to quantify wound healing/integration of the full-thickness skin graft were tissue oxygen saturation [StO_2_ (0–100%)] in the superficial skin layer (approximately 1 mm) and tissue hemoglobin index [THI as arbitrary units (0–100)]. To ensure that the 3D hyperspectral data cubes did not include information from deeper tissue layers, only the wavelength range between 520 and 580 nm was used to determine StO_2_. Briefly, a region of interest (ROI) in the shape of the respective flap surface was manually defined/plotted in the area of interest (Figure 3). The metric variables (StO_2_, and THI) automatically calculated herein are based on the spectral and spatial information per pixel. StO_2_ describes the relative oxygen saturation of the blood in the microcirculatory system in superficial tissue layers, records arterial and venous blood, and displays changes in oxygen supply and consumption directly on site in the tissue. Thus, StO_2_ represents the tissue oxygen saturation, which is mainly based on the blood volume in the venous part (75%) of the microcirculation and its oxygen saturation after delivery of oxygen to the tissue. The tissue oxygen saturation values of healthy volunteers are between 50–70% [15]. The THI describes the relative amount of hemoglobin in the microcircular system of the tissue area under consideration. This parameter gives an indication of inflow and/or outflow problems. Due to the different flap sizes and accessibility of the flaps, the area (pixel number) within the measurements and individuals varied accordingly. For calculation, one additional ROI around the flap served as a reference site and was located in the adjacent healthy skin (Figure 3). We have described the importance of the parameters and their combination for perfusion assessment in more detail in a previous paper [1].

IV. Clinical Monitoring

**Figure 4:**
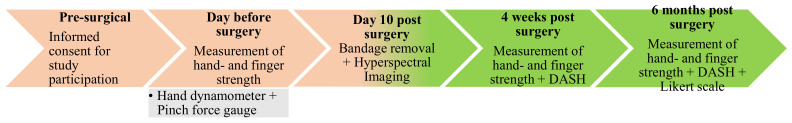
The time axis shows time points in the treatment of affected patients, as well as the parameters collected at each stage.

In order to evaluate the impact of RFFF and UFFF harvesting on hand function, we analyzed objective parameters such as both hand and finger force, as well as subjective patient assessments using the DASH questionnaire in German (DASH outcome measure, Institute for Work & Health (IWH), Toronto, SD, USA; https://dash.iwh.on.ca/about-dash; accessed on 2 January 2020). Eight questions in Likert scale format were used to assess patients’ subjective assessments of scarring (appearance) at the donor site, sensory impairment, and loss of grip strength, as well as whether any limitations affected their daily lives. The timing of data collection is shown in Figure 4. 

### 2.3. DASH Score

The Disabilities of the Arm, Shoulder and Hand (DASH) questionnaire is a 30-item questionnaire that helps to describe the disabilities experienced by people with upper-limb disorders and also to monitor changes in symptoms and function over time. To avoid misinterpretation of the results, patients were instructed to refer solely to the hand when answering the questions. This is of particular importance because a temporary or irreversible limitation of shoulder/arm mobility may occur due to a regularly performed neck dissection, which, however, has nothing to do with the RFFF or UFFF. Besides the first 30 questions about activities in everyday life, the DASH has two optional 4-item modules (Work and Sports (Performing Arts)) that can be used. Due to the severity of their underlying disease, older age, or adjuvant treatments, the patients included in this study generally did not participate in either sports or work activities after four weeks or six months, and therefore these two modules were not evaluated. Each item is scored with a 5-point scale ranging from 1 = no difficulty to 5 = unable. The results are summed and transformed into the DASH score in the following way:

$$\mathrm{DASH}=\frac{\mathrm{absolute}\;\mathrm{score}-30}{0.5}$$

The smallest amount of change that has to occur before the change can be considered a true change and not an error (Minimal Detectable Change (MDC)) is 12.75–17.23% according to the available literature [16]. The amount by which the score must change before it signifies an important or positive difference in the patient’s condition (Minimal Clinical Important Difference (MCID)) is 10.83–15 score points according to the available literature [17].

### 2.4. Scarring, Sensory and Functional Limitations

The subjective perception and evaluation of scarring at the donor site was assessed with four questions, subjectively experienced sensory disorders and loss of strength at the operated site, as well as the resulting limitations in everyday life, with two questions each. Participants were asked to rate the question number-scaled from 1 (strongly disagree) to 5 (strongly agree) (Table 1).

### 2.5. Grip Strength and Finger Pinch Strength

Grip strength was measured using a precision digital hand dynamometer (measurement range from 0 to 90 kg maximum, SAEHAN^®^, Saehan Corporation, Seoul, South Korea). Finger pinch strength during opposition movement (fingers I–V) was determined using a pinch force gauge (measurement range up to 27 kg, Baseline Evaluation Instruments, White Plains, NY, USA). The respective contralateral hand/fingers were also measured as an untreated control. Measurements were taken on the day before, as well as four weeks and six months after surgery.

### 2.6. Statistics

Raw data sets were saved in Excel^®^ sheets (Microsoft Corporation, Redmond, DC, USA) and subsequently transferred into SPSS Statistics^®^ (version 23.0.0.2, MacOS X; SPSS Inc., IBM Corporation, Armonk, NY, USA). Data were expressed as mean (m), standard deviation (SD±), minimum (min), and maximum (max). Qualitative variables were summarized as counts and percentages. In addition to the descriptive analysis, the dependency analysis included tests to detect/exclude differences and correlations. Results were analyzed for statistical significance by the use of analysis of variance (ANOVA(#)), unpaired non-parametric-Mann–Whitney-U-tests ($), and Students’ *t*-test (*). To investigate whether the means of several dependent samples differ, a Wilcoxon matched-pairs signed rank test (**) was performed. Correlations between two categorical variables were tested using the Pearson Chi-Square Test (^+^), or, in the case of expected cell frequencies < 5, using Fisher’s Exact Test (^++^). *p*-values of ≤0.05 were termed significant. Line charts with plotted means, dot plots, and bar charts were used for illustration purposes.

## 3. Results

### 3.1. Demographics

A total of 30 patients (17 male and 13 female) with a mean age of 67 years (34–85 years) were included in this prospective clinical, randomized controlled comparative study. The ratio of RFFF and UFFF was 1:1. General patient demographics are listed in Table 2. In all patients, flap transfer was carried out to reconstruct intraoral defects resulting from surgical tumor therapy of oral squamous cell carcinoma.

### 3.2. Flap Success/Performance

In this study, no complications requiring revision occurred; thus, the success rate was 100%.

#### 3.2.1. Hyperspectral Flap Perfusion Monitoring

##### Tissue Oxygenation Saturation (StO_2_)

**Figure 5:**
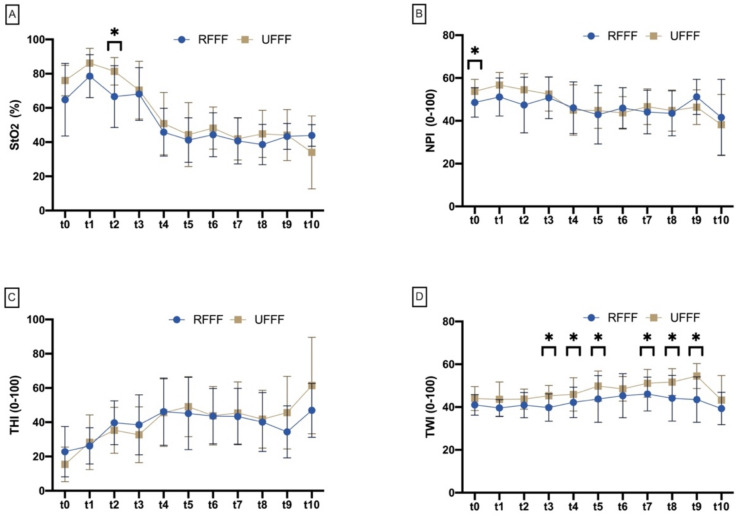
Connected plot diagram shows mean and standard deviation of (**A**) tissue oxygen saturation (StO_2_), (**B**) near infrared perfusion index (NPI), (**C**) relative tissue hemoglobin (THI), and (**D**) tissue water index (THI) in comparison of radial forearm free flap (RFFF) and ulnar forearm free flap (UFFF). Asterisks mark measurement time points with significant differences between RFFF and UFFF.

Comparing RFFF and UFFF, StO_2_ of the forearm flap revealed a similar trend from pre-harvest (baseline), through liberalization, anastomosis, insertion, and three-day follow-up. A significant difference between RFFF and UFFF was seen after anastomosis only (*p* = 0.017), with higher oxygenation in UFFF (Figure 5).

##### Near Infrared Perfusion Index (NPI)

Near-infrared perfusion index (NPI) also revealed similar characteristics between RFFF and UFFF over time; however, it showed significantly higher values in UFFF at the baseline/origin (53.8 ± 5.6 vs. 48.6 ± 6.9; *p* = 0.035) (Figure 5).

##### Tissue Hemoglobin Index (THI)

For tissue hemoglobin index (THI), as a surrogate parameter of microcirculation blood flow, there were no significant differences in the comparison of RFFF and UFFF at any time point of measurement (Figure 5).

##### Tissue Water Index (TWI)

Tissue water index between RFFF and UFFF differed neither preoperatively, nor after liberalization or anastomosis. After flap insertion (*p* = 0.008), as well as at the time periods of 0–12 h (*p* = 0.046), 12–24 h (*p* = 0.034), 36–48 h (*p* = 0.017), 48–60 h (*p* = 0.011), and 60–72 h (*p* = 0.005), there were significantly higher values in the group after UFFF transfer (Figure 5).

##### Δ Reference

**Figure 6:**
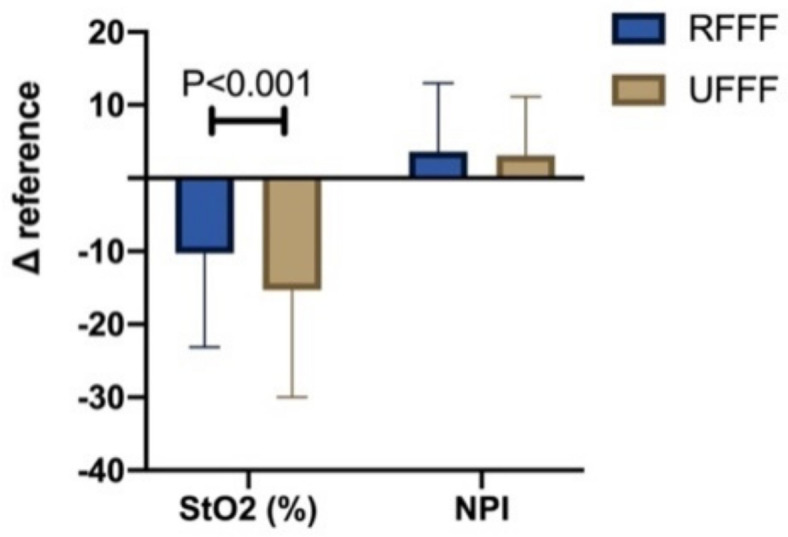
Bar chart showing the difference of StO_2_ and NPI between flap surface and adjacent reference site in comparison of RFFF and UFFF.

Independent of the time point/interval, StO_2_ difference between flap surface and reference area (resident tissue) StO_2_ Δ reference was significantly (*p* < 0.001) increased in UFFF (−15.3% ± 14.7%) compared with RFFF (−10.3% ± 12.8%). No significant difference was found for NPI (*p* = 0.216) (Figure 6).

### 3.3. Donor Site Morbidity

#### 3.3.1. Thenar and Hypothenar Eminence–HSI Perfusion Monitorin

##### Tissue Oxygen Saturation (StO_2_)

**Figure 7:**
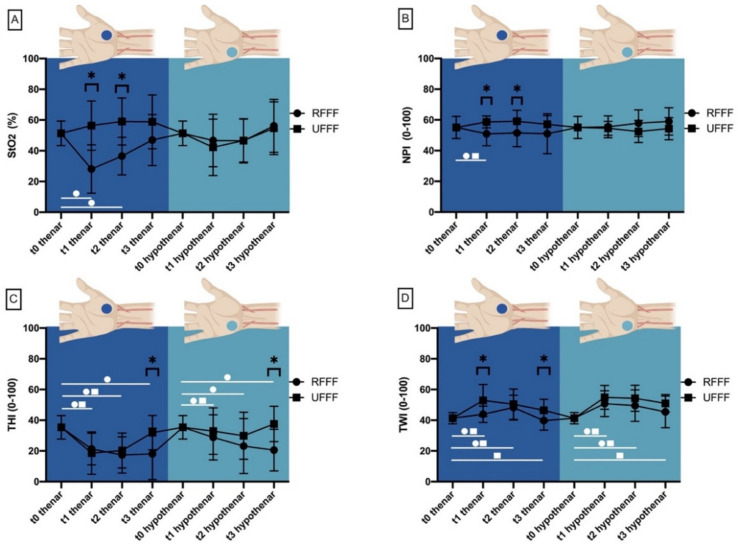
Plot diagram showing (**A**) tissue oxygenation saturation (StO_2_), (**B**) near perfusion index (NPI), (**C**) tissue hemoglobin (THI) and–(**D**) water index (TWI) of the thenar and hypothenar eminence at different measurement time points after RFFF and UFFF transfer. Asterisks mark measurement time points with significant differences between RFFF and UFFF.

Preoperatively, tissue oxygenation saturation (StO_2_) at the thenar (ROI radial) and hypothenar eminence (ROI ulnar) was similar (51.3 ± 8%). After 10 days (t1), RFFF–StO_2_ was significantly decreased (28.1 ± 15.8%) at the thenar eminence when compared with UFFF (56.3 ± 16.1%; *p* < 0.001). Compared with baseline (t0), RFFF–StO_2_ was also significantly decreased (*p* = 0.003). After 4 weeks (t2), thenar StO_2_ still differed significantly between RFFF (36.5 ± 12.2%) and UFFF (59.1 ± 15.3%; *p* < 0.001). Compared with baseline (t0), RFFF–StO_2_ was significantly decreased (*p* = 0.006). There were no differences between RFFF and UFFF at the hypothenar eminence at any time point (Figure 7).

##### Near Infrared Perfusion Index (NPI)

Preoperatively, the near infrared perfusion index (NPI) at the thenar (ROI radial) and hypothenar eminence (ROI ulnar) was similar (55.1 ± 7.2). At t1, thenar NPI differed significantly between RFFF (50.9 ± 7.8) and UFFF (58.7 ± 3.9; *p* < 0.001). Compared with baseline (t0), RFFF–NPI was also significantly decreased (*p* = 0.048), whereas UFFF–NPI was increased (*p* = 0.012). After 4 weeks (t2), thenar NPI was still significantly (*p* < 0.0.21) higher in UFFF (51.5 ± 13) compared with RFFF (59.1 ± 7.1). There was no difference in NPI between RFFF and UFFF in the hypothenar eminence at any time point.

##### Tissue Hemoglobin Index (THI)

Preoperatively, the tissue hemoglobin index (THI) at the thenar (ROI radial) and hypothenar eminence (ROI ulnar) was similar (35.4 ± 7.6). Except for t3, at both the thenar (18.1 ± 16.7 vs. 31.9 ± 11.2; *p* = 0.014) and hypothenar eminence (20.6 ± 13.6 vs. 37.6 ± 11.5; *p* = 0.009), THI did not differ significantly between RFFF and UFFF. At the thenar, THI decreased significantly compared with the baseline at t1 and t2 for both RFFF (*p* = 0.001) and UFFF (*p* = 0.003, *p* < 0.001). At t3, THI was significantly decreased in RFFF only (*p* = 0.022). At hypothenar, THI decreased significantly (*p* < 0.001, *p* = 0.048, *p* = 0.022) compared with the baseline at t1, t2, and t3 with RFFF. In patients with UFFF, the only significant decrease occurred by t1 (*p* < 0.001).

##### Tissue Water Index (TWI)

Preoperatively, the tissue water index (TWI) at the thenar (ROI radial) and hypothenar eminence (ROI ulnar) was similar (41.3 ± 3.7). At the thenar eminence, TWI was significantly higher in UFFF (52.9 ± 10.4 and 46.5 ± 7.2) than in RFFF (43.9 ± 5.3 and 39.8 ± 6.2) both 10 days (t1; *p* = 0.006) and 6 months (t3; *p* = 0.030) after surgery. In contrast, there was no significant difference between RFFF and UFFF at the hypothenar area (ROI ulnar) at any timepoint. At the thenar, TWI increased significantly compared with the baseline at t1 and t2 for both RFFF (*p* = 0.041, *p* = 0.005) and UFFF (*p* = 0.001, *p* = 0.005). At t3, TWI was significantly increased in UFFF only (*p* = 0.034). At hypothenar, TWI increased significantly compared with the baseline at t1 and t2 for both RFFF (*p* = 0.004) and UFFF (*p* < 0.001). At t3, TWI was significantly increased in UFFF only (*p* = 0.003) (Figure 7).

#### 3.3.2. Quantification of Wound Healing Dynamics

##### Baseline (t0)

**Figure 8:**
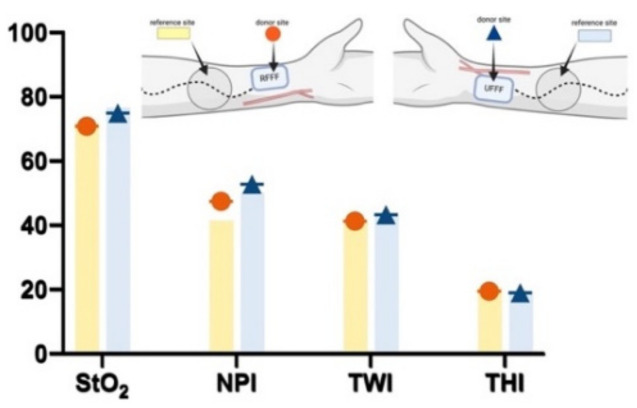
Bar chart shows hyperspectral wound healing characteristics (StO2, NPI, TWI and THI) preoperatively/baseline (t0) at the donor site (colored symbol) and neighboring reference site (colored bar/column). The red circle represents RFFF and the dark blue triangle represents UFFF.

Prior to flap preparation (t0/baseline), there was no significant difference in StO_2_, NPI, TWI, and THI in a comparison of RFFF and UFFF. When comparing the projected donor and untreated neighbored reference sites, there was no significant difference in StO_2_, NPI, TWI, and THI for RFFF or for UFFF (Figure 8).

##### Ten Days after Surgery (t1)

**Figure 9:**
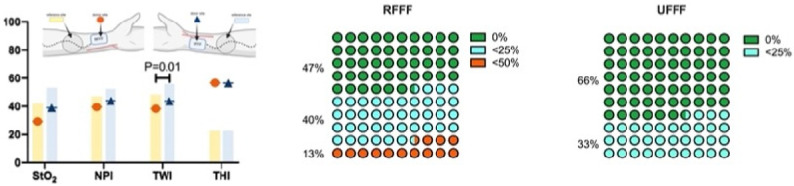
Bar chart shows hyperspectral wound healing characteristics (StO_2_, NPI, TWI and THI) 10 days after surgery (t1) at the donor site (colored symbol) and neighboring reference site (colored bar/column). The red circle represents RFFF and the dark blue triangle represents UFFF.

On the tenth postoperative day (t2), StO_2_, NPI, and THI at the donor and reference sites were not different when comparing RFFF and UFFF, whereas the tissue water index (TWI) was significantly higher in UFFF, both at the donor and the reference site (*p* = 0.01; Figure 9). Compared with the reference site, StO_2_, NPI, and TWI were significantly decreased in the donor region of both RFFF (*p* < 0.001, *p* = 0.027, *p* < 0.001) and UFFF (*p* < 0.001, *p* = 0.004, *p* < 0.001). In contrast, THI was increased at the donor site (*p* < 0.001) (Figure 9). Among the patients, 47% (*n* = 7) in the RFFF group experienced regular wound healing, 40% (n = 6) presented with impaired healing over an area ≤ 25%, and 13% (n = 2) presented with impaired healing over an area ≤ 50% of the donor site. Thus, impaired wound healing was found in 53% of patients with RFFF. In the UFFF group, 67% (n = 10) presented with regular wound healing, and 33% showed impaired wound healing over an area ≤ 25% of the donor site. More severe cases of impaired healing did not occur (Figure 9). We were not able to reject the null hypothesis that incidence of wound healing disorders and flap type are independent (Fisher’s Exact Test = 0.462; Phi = 0.269; Cramer’s V = 0.269). Thus, the two parameters are assumed to be independent.

##### Four Weeks after Surgery (t2)

**Figure 10:**
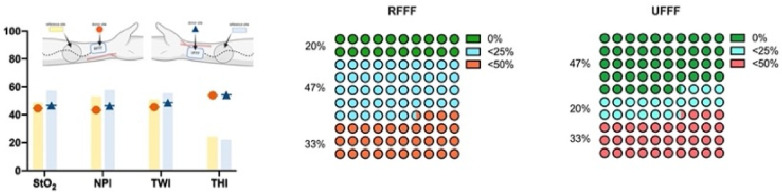
Bar chart shows hyperspectral wound healing characteristics (StO2, NPI, TWI and THI) 4 weeks after surgery (t1) at the donor site (colored symbol) and neighboring reference site (colored bar/column). The red circle represents RFFF and the dark blue triangle represents UFFF.

Four weeks postoperatively (t3), StO_2_, NPI, TWI, and THI were not significantly different at either the donor site or the neighboring reference region when comparing RFFF and UFFF. Compared with the reference site, StO_2_, NPI, and TWI were significantly decreased in the donor region of both RFFF (*p* = 0.011 *, *p* = 0.023 *, *p* < 0.001 *) and UFFF (*p* = 0.005 **, *p* = 0.005 **, *p* < 0.001 **) (Figure 10). In contrast, THI was still significantly increased at the donor site of both RFFF (*p* = 0.011 *) and UFFF (*p* = 0.005 **). In the RFFF group, 20% (n = 3) of patients presented with regular wound healing at the donor site, 47% (n = 7) with impaired healing over an area ≤ 25%, and 33% (n = 5) with impaired healing over an area ≤ 50% of the donor site. Among the UFFF group, 47% (n = 7) of patients presented with a regular wound healing situation, 20% (n = 3) presented with impaired wound healing over an area ≤ 25%, and 33% (n = 5) presented with wound healing impairment over an area ≤ 50% of the donor site (Figure 10). Overall, impaired wound healing was found in 80% of RFFF and in 53% of UFFF. We were not able to reject the null hypothesis that incidence of wound healing disorders and flap type are independent (Fisher’s Exact Test = 0.245; Phi = 0.121; Cramer’s V = 0.121). Thus, the two parameters are assumed to be independent.

##### Six Months after Surgery (t3)

**Figure 11:**
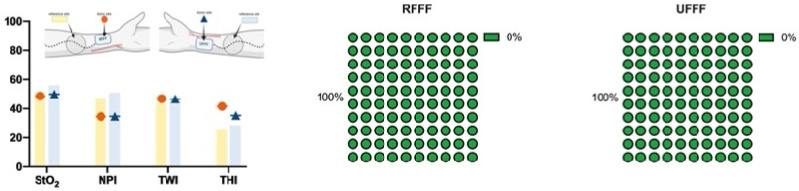
Bar chart shows hyperspectral wound healing characteristics (StO2, NPI, TWI, and THI) 6 months after surgery (t1) at the donor site (colored symbol) and neighboring reference site (colored bar/column). The red circle represents RFFF and the dark blue triangle represents UFFF.

Six months after surgery, there was no significant difference between RFFF and UFFF regarding StO_2_, NPI, TWI, and THI at the donor site or in the neighboring reference region. Compared with the RFFF reference site, NPI still appeared significantly decreased at the donor site (*p* = 0.003), whereas THI was still increased (*p* = 0.013). Compared with UFF reference sites, both Tissue oxygenation saturation (StO_2_) and NPI were significantly decreased (*p* = 0.005, *p* = 0.002) at the UFFF donor site. There was no area with impaired wound healing at the donor site in either RFFF or UFFF (Figure 11).

#### 3.3.3. Quality of Life Analysis

When assessing DASH scores on postoperative day 28 (DASH 1), as well as six months (DASH 2) after surgery, there was significant difference within the RFFF group (*p* = 0.006). No significant difference was present within the UFFF group (*p* = 0.865). There was no difference between RFFF and UFFF in DASH 1 at 4 weeks (*p* = 0.191) and DASH 2 at 6 months (*p* = 0.803). For RFFF, the difference between DASH 1 and DASH 2 was −30% on average, whereas it was −3% for UFFF. When comparing RFFF and UFFF, the difference is significant (*p* = 0.005). The subjective evaluation (Likert scale) on the donor sites’ esthetics, palm sensitivity, and associated limitations due to sensitivity disorders, subjective grip force reduction, and associated limitations, revealed no difference in a comparison of RFFF and UFFF (Table 3).

#### 3.3.4. Grip and Finger Pinch Strength

Within the RFFF group, there was a significant reduction (*p* = 0.019) in grip strength at the donor site after four weeks compared to presurgical, which had, however, mostly recovered (*p* = 0.424) after six months. In contrast, the UFFF group revealed a significant decrease in strength both after four weeks (*p* = 0.003) and after six months (*p* = 0.008). For RFFF, loss of grip strength after 6 months was only 1.7 %. In contrast, loss of grip strength following UFFF transfer was 13% between presurgical and 6 months. The difference was not significant when comparing RFFF and UFFF (*p* = 0.57). There was no difference in grip strength between RFFF and UFFF, neither presurgical, nor after 4 weeks or 6 months. On the contralateral control side, there was no significant difference either within or between RFFF and UFFF (Table 4).

**Figure 12:**
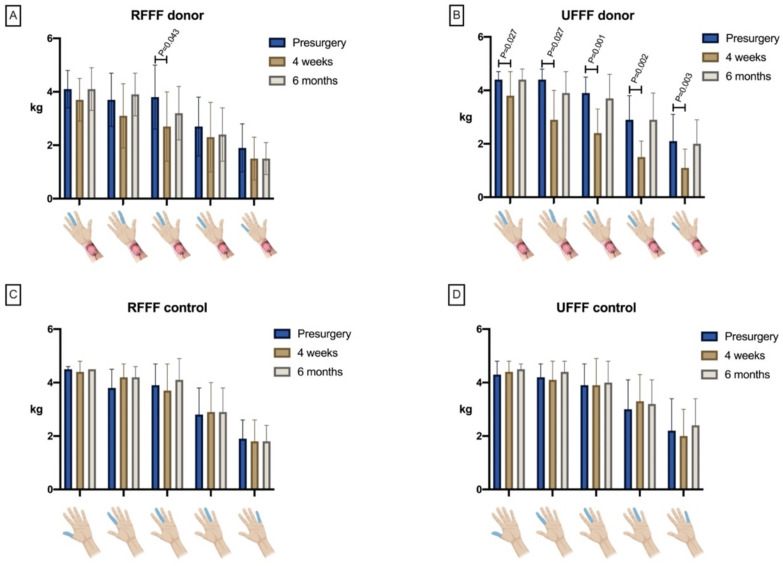
Bar charts show mean pinch strength of RFFF and UFFF donor site (**A**,**B**) and control site (**C**,**D**) preoperatively, and 4 weeks and 6 months postoperatively over time.

When comparing the RFFF donor and contralateral control site, there was a significant difference in pinch force of the thumb (finger 1) and index finger after four weeks, the middle finger (finger 3) after four weeks and six months, and the small finger (finger 5) after six months (Table 5). Compared to the baseline, there was a significantly decreased pinch force of the middle finger at four weeks after surgery only (*p* = 0.043). After six months, however, strength returned to normal (*p* = 0.102) (Figure 12). On the contralateral control side, there was no significant change in force for any finger; although not significant, there was a trend toward increasing pinch strength in all fingers of the control hand (Figure 12C,D). In a comparison of the UFFFs’ surgical and non-surgical site (control), strength was found to be significantly decreased in the thumb (finger 1) after four weeks, in the index finger (finger 2) after four weeks and six months, in the middle finger (finger 3) after four weeks, in the ring finger (finger 4) after four weeks and six months, and in the small finger (finger 5) after four weeks (Table 6). Compared to the baseline, there was a significant decrease in the strength of the thumb at four weeks after surgery (*p* = 0.027), which, however, returned to normal after six months (*p* = 0.655; Table 6). The same changes were found for fingers two (*p* = 0.004, *p* = 0.091), three (*p* = 0.001, *p* = 0.065), four (*p* = 0.002, *p* = 0.532), and five (*p* = 0.003, *p* = 0.217; Figure 12).

## 4. Discussion

The aim of this prospective randomized controlled trial was to compare RFFF and UFFF in terms of success rates, donor site morbidity, and quality-of-life, as well as objective functional and quantitative wound healing parameters. The 30 patients included matched the population of other studies in terms of demographics and specific medical history [9,10].

Forearm free flap transfer has been used for years and remains a well-established procedure for microsurgical defect reconstruction in the head and neck region. Of particular advantage is the flap anatomy (thin flap, long and caliber-strong vascular pedicle), as well as the possibility of simultaneous flap raising during tumor-ablative procedures or recipient vessel exposure. Donor site morbidity has a pivotal influence on flap selection for reconstruction of soft-tissue defects. There is controversy in the literature concerning which of the two flaps (ulnar or radial) should be preferred, with some studies describing the superiority of the UFFF due to a lower donor site morbidity, less hairy skin, and a comparable success rate [8,9,10]. With hyperspectral perfusion monitoring, levels of StO_2_, NPI, TWI, and THI were consistent with those of previous studies published by our group investigating the characteristics of normal and minor perfused flaps at the measurement time points t0 (origin/baseline) to t10 (>72 h after surgery) [1,13]. Similarly, the value difference between flap surface and adjacent reference measurement site (Δ reference) for StO_2_ and NPI was comparable to previous results, confirming the reproducibility and overall procedural quality of the hyperspectral perfusion monitor [13]. The historical assumption of the ulnar artery as the dominant blood supply to the hand and the earlier description of the RFFF have given way to a preference for the RFFF over the UFFF when considering forearm tissue for use in reconstructive surgeries [18]. Keen et al. observed that the ulnar artery is larger than the radial artery in the proximal forearm; however, the radial artery has a greater diameter at the wrist level [19]. Coleman and Anson demonstrated that there was no anastomosis between the superficial palmar arch and the radial artery in 12–60% of cases, and that there was no anastomosis between the deep palmar arch and the ulnar artery in 54% [20,21]. If both anastomoses were not present, resection of one artery would theoretically result in a critical reduction in perfusion of the affected hand. Using detailed radioisotopic analysis, it was possible to demonstrate the dominant role of the radial artery in the blood supply to the hand [21,22]. In contrast, de Vincente et al. demonstrated no difference in hand perfusion between the radial and ulnar artery following RFFF and UFFF using Doppler ultrasound and plethysmography [21].

In the present study, we assessed hand perfusion at the thenar and hypothenar eminence using hyperspectral imaging (HSI), assessing StO_2_, NPI, THI, and TWI. The StO_2_ value determined by HSI reflects the percentage hemoglobin oxygen saturation in the capillary region of the tissue microcirculation. Thus, HSI indicates changes in oxygen supply and consumption directly on site in the tissue. StO_2_ is generally lower than arterial oxygen saturation (SaO_2_) or pulse oximetry oxygen saturation (SpO_2_) and is intermediate between arterial and venous oxygen saturation (SvO_2_). The SpO_2_ value measured by pulse oximeter is the most commonly used measure of hemoglobin oxygen saturation. Unlike the StO_2_ value, the SpO_2_ value is calculated from the pulsatile (arterial) portion of the rapidly sampled measurement signal, so this value is considered a measure of arterial oxygen saturation. However, this provides useful information on lung function, but gives no information about oxygen supply to the tissue or about oxygen uptake by organs, as is the case with hyperspectral imaging. In models, 70% venous and 30% arterial oxygen saturation is often used to calculate current tissue oxygen saturation [15]. StO_2_ in the thenar eminence was found to be significantly decreased after RFFF transfer up to 4 weeks after surgery compared with the baseline. Regarding NPI, this was true for RFFF and UFFF until postoperative day 10 (t1). Only the tissue hemoglobin index (THI), as an indirect parameter for blood flow (70% venous), was significantly decreased at 6 months after RFFF transfer, both at thenar and hypothenar eminence.

One of the most fundamental but powerful clinical tools when managing patients with wounds is visual observation. Unfortunately, inspection requires significant experience or training [23]. Inspection poses difficulties when parameters do not change typically, e.g., in the case of weak or nontypical signs of infection in chronic wounds. Hyperspectral imaging provides information on microcirculation and tissue oxygenation, i.e., tissue hemoglobin oxygen saturation (StO_2_) (oxygenation index (in %) of tissue for superficial perfusion), near infrared perfusion index (NPI), and for deeper perfusion and tissue hemoglobin index (THI) as the percental volume of hemoglobin presenting superficial blood flow. In this study, the healing dynamics of the full-thickness skin graft used for coverage were characterized by hyperspectral imaging, and the incidence and extent of wound healing disorders were documented. Thereby, oxygenation and blood flow of the donor site were found to be equal in a comparison of RFFF and UFFF preoperatively (t0). From postoperative day 10, through follow-up at 4 weeks and 6 months, oxygenation, perfusion, and water content (StO_2_, NPI, THI, TWI) were found to be equally distributed at the donor site and the untreated surrounding reference in RFFF and UFFF. In this process, oxygenation and perfusion constantly increased at the donor site (full-thickness skin graft), which was significantly inferior compared with the surrounding healthy skin at the beginning and reached almost equal levels after six months.

Regarding wound healing, on postoperative day ten, 53% of patients after RFFF but only 33% of patients after UFFF transfer demonstrated impaired wound healing at the donor site. At the 4-week follow-up, 80% of patients after RFFF transfer but only 53% of patients after UFFF transfer presented with impaired wound healing at the donor site. For RFFF, the affected area was <25% in 47% of patients and ≤50% in 33% of patients. For UFFF, the affected area was ≤25% in 20% of patients and ≤50% in 33% of patients. However, this study demonstrated no statistical correlation between the incidence of wound healing disorders and flap type (RFFF versus UFFF). After six months, wound healing in the sense of a closed skin surface was completed in both groups. In accordance with this, Chio et al. reported wound healing complications (defined as skin graft failure and/or tendon exposure) in up to 80% of patients at four weeks after RFFF. As in our study, there was an increase in the complication rate from the first (after the first follow-up wound dressing change) to the second follow-up time point (after 4 weeks) [24]. For RFFF, Schwarzer et al. reported an overall complication rate of 38% in their population [25]. In contrast, Bertino et al. revealed no wound healing disturbances in the area of both the RFFFs’ and UFFFs’ donor sites following closure with a full-thickness skin graft from the groin after 29 days [6]. Sieg et al. directly compared outcomes of the UFFF to the RFFF and noted decreased donor site morbidity after UFFF transfer in addition to decreased rates of dehiscence [8]. A probable reason for the different incidence of wound healing complications results from its divergent definition (e.g., minimum size for partial and total necrosis, tendon exposure, dehiscence, hematoma, seroma, infection).

There was a significant (*p* = 0.006) decrease in the DASH score (improvement in subjectively experienced dysfunction) from 4 weeks to 6 months in the RFFF group (−29%). In contrast, there was no relevant improvement in the UFFF group during this period. Although the rate of improvement compared between RFFF and UFFF was significantly different (*p* = 0.005) in favor of RFFF, the mean DASH score after 6 months differed only slightly between RFFF and UFFF (29 ± 15 vs. 32 ± 24). With -29% and thus >17.23% (MDC), the difference can be considered a real change according to the relevant literature [16]. At the same time, the decrease in DASH with –14 points and thus within the range of MCID (10.83–15 points) can be considered a significant improvement of the patient’s condition [17]. In contrast, Bertino et al. demonstrated mean DASH scores of 7 after RFFF and 10 after UFFF transfer. However, there is no information on when the DASH score was assessed, as this was a retrospective study. This is crucial, since assessing the score on the basis of patient memories would have an unpredictable impact on the results. Riecke et al. reported an increase in DASH by 16 points (+35.5%) 3 months after RFFF transfer. However, the mean values are not given, and in contrast to our study, the comparison was made to the preoperative status, which limits the comparability of the results [26]. However, the study by Sieg et al. confirms our results with a constant decrease of subjectively experienced dysfunction over time. They demonstrated a DASH score of 21.5 after 12 months following UFFF transfer, a score of 18.2 between 12 and 36 months, and a score of 9.2 > 36 months after UFFF transfer [9]. Regarding the aesthetic experience of the donor site, patients after RFFF transfer were neither satisfied nor dissatisfied (Likert = 3) on average, whereas patients after UFFF transfer were rather satisfied with the result (Likert = 4). One patient (6.7%) indicated strong dissatisfaction with the esthetic outcome after RFFF, whereas 8 patients (53%) were rather satisfied or satisfied with the outcome. In the UFFF group, one patient (6.7%) was very and one (6.7%) was rather unsatisfied with the esthetic outcome, whereas 10 patients (67%) were rather or very satisfied with the result. These results are consistent with the current literature as the minority of patients after RFFF or UFFF transfer are dissatisfied with the aesthetic outcome at the donor site [10,27]. In accordance with this, Molteni et al. reported that patient satisfaction depends on the type of skin graft used for coverage (split versus full thickness), with the local full-thickness skin graft found to be superior [28]. Subjective assessment of sensitivity related to the hand of the donor site revealed hypoaesthesia in 27% of patients after RFFF transfer and in 47% of patients after UFFF transfer. Of these, 20% from the RFFF group and 33% from the UFFF group reported hypoesthesia-related limitations in everyday life. Regarding the RFFF, our results are consistent with the literature, as an average of 27% of the patients reported subjective sensory loss in the palm area during follow-up [27]. In contrast, Riecke et al. and Schwarzer et al. described increased rates of dysaesthesia in their population (40% and 56%, respectively) [25,26]. In contrast to the present results, Sieg et al. found 18% of patients with impaired sensitivity after UFFF transfer at 3 years; however, only 6% reported dysaesthesia after 6 years. Since experience suggests a reduction in subjective sensitivity disorders over time, or rather that affected patients perceive them less intensely, we also expect a decrease in perceived hypoesthesia in our patients over time. The subjective evaluation of grip strength via Likert scale revealed a decrease in strength in 40% of RFFF patients compared with 60% of UFFF patients. These results are consistent with those of Chambers et al. who observed a slight reduction in grip strength in 90.5% of patients after RFFF transfer. Comparable studies on subjective strength rating after UFFF transfer are not available. In contrast, the objective measurement data of the present study revealed grip strength reductions of 2% after RFFF and of 13% after UFFF transfer at 6 months after surgery. For RFFF, these results are consistent with the study results of Brown et al. [29], but they demonstrate a slightly lower average loss of strength (0.1 versus 3 kg) [27]. RFFF measurements of individual pinch strength revealed a significant reduction in the middle finger after 4 weeks, which, however, recovered after 6 months. In contrast, UFFF showed a significant reduction in strength in all five fingers after 4 weeks, but this normalized after 6 months on all fingers. Especially after 6 months, these results are in contrast with the literature, considering that an average force loss of 1 kg was registered after RFFF transfer in the key pinch test [27]. In this regard, the data refer to a systematic review, with only three studies reporting relevant information on pinch strength. Witt et al. reported a significant reduction in pinch strength with RFFF compared with the control hand, although these results should be interpreted critically, since, as also shown in our study, pinch strength on the control side appears to increase due to a constant increase in load during immobilization of the donor site [30].

## 5. Conclusions

RFFF and UFFF both demonstrate similar success rates and flap performance with good perfusion dynamics after flap transfer. However, the overall difference of StO_2_ from the neighboring reference tissue was significantly increased in UFFF compared with RFFF. With regard to donor site morbidity, there was a significant reduction in blood flow (THI) in the thenar and hypothenar eminence 6 months after RFFF transfer, with no difference between RFFF and UFFF in superficial and deep tissue oxygenation. In contrast to the literature, there was no difference between RFFF and UFFF regarding wound healing disorders, grip and pinch strength, and subjective disability in daily life (DASH), neither after 4 weeks nor after 6 months. Thus, both flaps can be equally recommended for the reconstruction of head and neck defects.

## Figures and Tables

**Table 1:** Likert scale for rating aesthetics, sensitivity and grip strength following RFFF and UFFF transfer as well as their impact on daily life.

	Strongly Disagree	Somewhat Disagree	Neither Agree nor Disagree	Somewhat Agree	Strongly Agree
**1. Are you satisfied with the shape of the scar ar your forearm?**	1	2	3	4	5
**2. Are you satisfied with the size of the scar at your forearm?**	1	2	3	4	5
**3. Are you satisfied with the color of the scar at your forearm?**	1	2	3	4	5
**4. Are you satisfied with the thickness of the scar at your forearm?**	1	2	3	4	5
**5. Do you feel any sensory disturbances in the area of the donor sites’ palm since surgery?**	1	2	3	4	5
**6. If yes, does it limit your everyday life?**	1	2	3	4	5
**7. Do you experience any loss of grip strength on the donor site at this time?**	1	2	3	4	5
**8. If yes, does it limit your everyday life?**	1	2	3	4	5

**Table 2:** Patient demographics.

	Total	RFFF	UFFF
N	30	15	15
**Age**	66.7 ± 10.4	67.6 ± 11.7	65.9 ± 9.3
**Gender**			
Male	17 (57%)	8 (53%)	9 (60%)
Female	13 (43%)	7 (47%)	6 (40%)
**Tumor site**			
Tongue	5 (17%)	2 (13%)	3 (20%)
Mouth floor	7 (23%)	3 (20%)	4 (27%)
Buccal	8 (27%)	6 (40%)	2 (13%)
Hard palate	1 (3%)	0	1 (6%)
Soft palate	1 (3%)	0	1 (6%)
Alveolar crest	8 (27%)	4 (27%)	4 (27%)
**Neck Dissection**			
Bilateral	27 (90%)	14 (93%)	13 (87%)
Ipsilateral	2 (7%)	0	2 (13%)
Contralateral	1 (3%) (2nd cancer)	1 (7%) (2nd cancer)	0
**Adjuvant radiotherapy**			
Yes	20 (67%)	11 (73%)	9 (60%)
No	10 (33%)	4 (27%)	6 (40%)
**Recipient vessel (artery)**			
Superior thyroid	22 (73%)	11 (73%)	11 (73%)
Lingual	4 (13%)	2 (13%)	2 (13%)
External carotid	2 (7%)	1 (7%)	1 (7%)
Facial	2 (7%)	1 (7%)	1 (7%)
**Recipient vessel (vein)**			
Internal jugular vein	30 (100%)	15 (50%)	15 (50%)
**Flap size (cm^2^)**	34.3 ± 12.7	37.6 ± 16.4	30.90 ± 6.4
**Duration of surgery (minutes)**	482 ± 102	494 ± 122	467 ± 80

**Table 3:** DASH Score and Likert Scale in comparison between RFFF and UFFF. (*) indicates significance.

	Total	RFFF		UFFF		
	Mean ± SD	Mean ± SD	Δ DASH 1–2 (%); *p*-Value	Mean ± SD	Δ DASH 1–2 (%); *p*-Value	*p*-Value
DASH 1 4 weeks	38 ± 22	43 ± 19	−30%; 0.006 *	33 ± 24	−3%; 0.865	0.191
						0.005 *
DASH 2 6 months	30 ± 19	29 ± 15		32 ± 24		0.803
**Likert**						
Scar	3 ± 1	3 ± 1		4 ± 1		0.766
Sensitivity	3 ± 1	3 ± 1		3 ± 1		0.120
Handicap by < sensitivity	3 ± 1	2 ± 1		3 ± 1		0.432
Grip force	3 ± 1	3 ± 1		3 ± 1		0.266
Handicap by < grip force	3 ± 1	3 ± 1		3 ± 1		0.413

**Table 4:** Grip force (pound) in comparison between RFFF and UFFF before, four weeks and six months after surgery. Asterisks mark significant differences. Right column shows the statistical differences between RFFF and UFFF.

	Grip Force	Total	RFFF		UFFF		RFFF vs. UFFF
		Mean ± SD	Mean ± SD	*p*-Value	Mean ± SD	*p*-Value	*p*-Value
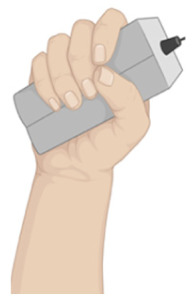	Donor presurgical	27.5 ± 8.9	23.9 ± 8.3		30.9 ± 8.2		0.058
	Donor 4 weeks	18.3 ± 8.5	17.8 ± 9.9	0.019 *	18.7 ± 7.3	0.003 *	0.830
	Donor 6 months	25.3 ± 9.1	23.9 ± 9.1	0.424	26.8 ± 9.3	0.008 *	0.562
	Control pre surgical	29.3 ± 8.6	28.1 ± 8.3		30.4 ± 9.1		0.402
	Control 4 weeks	29.9 ± 9.4	28.7 ± 10.6	0.807	31.2 ± 8.5	0.279	0.650
	Control 6 months	30.9 ± 7.5	29.2 ± 8.2	0.594	32.6 ± 6.7	0.894	0.193

**Table 5:** Demonstrates mean pinch strength after RFFF transfer comparing donor site and contralateral control site. Asterisks mark significant results.

RFFF Surgery	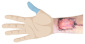			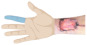			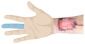			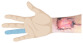			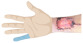		
	Pre Surgery	4 we.	6 mo.	Pre Surgery	4 we.	6 mo.	Pre Surgery	4 we.	6 mo.	Pre Surgery	4 we.	6 mo.	Pre Surgery	4 we.	6 mo.
Mean ± SD	4.1 ± 0.7	3.7 ± 0.8	4.1 ± 0.8	3.7 ± 1.0	3.1 ± 1.2	3.9 ± 0.8	3.8 ± 1.2	2.7 ± 1.3	3.2 ± 1.0	2.7 ± 1.1	2.3 ± 1.3	2.4 ± 1.0	1.9 ± 0.9	1.5 ± 0.8	1.5 ± 0.6
*p*-value	0.18	0.012 *	0.068	0.713	0.008 *	0.175	0.715	0.021 *	0.015 *	0.893	0.075	0.057	0.917	0.136	0.014 *
Mean ± SD	4.5 ± 0.1	4.4 ± 0.4	4.5 ± 0	3.8 ± 0.7	4.2 ± 0.5	4.2 ± 0.4	3.9 ± 0.8	3.7 ± 1.0	4.1 ± 0.8	2.8 ± 1.0	2.9 ± 1.1	2.9 ± 0.9	1.9 ± 0.7	1.8 ± 0.8	1.8 ± 0.6
**RFFF** **Control**	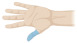			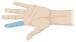			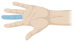			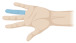			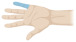		

**Table 6:** Demonstrates mean pinch strength after UFFF transfer comparing donor site and contralateral control site. Asterisks mark significant results.

UFFF Surgery	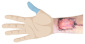			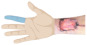			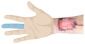			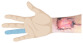		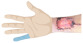			
	Pre Surgery	4 we.	6 mo.	Pre Surgery	4 we.	6 mo.	Pre Surgery	4 we.	6 mo.	Pre Surgery	4 we.	6 mo.	Pre Surgery	4 we.	6 mo.
Mean ±SD	4.4 ± 0.3	3.8 ± 0.9	4.4 ± 0.4	4.4 ± 0.4	2.9 ± 1.1	3.9 ± 0.8	3.9 ± 0.6	2.4 ± 0.9	3.7 ± 0.9	2.9 ± 0.9	1.5 ± 0.6	2.9 ± 1.0	2.1 ± 1.0	1.1 ± 0.7	2.0 ± 0.9
*p*-value	0.180	0.011 *	0.317	0.216	0.002 *	0.043 *	0.811	<0.001 *	0.232	0.430	<0.001 *	0.050 *	0.388	0.005 *	0.123
Mean ±SD	4.3 ± 0.5	4.4 ± 0.4	4.5 ± 0.2	4.2 ± 0.5	4.1 ± 0.7	4.4 ± 0.4	3.9 ± 0.8	3.9 ± 1.0	4.0 ± 0.8	3.0 ± 1.1	3.3 ± 1.0	3.2 ± 0.9	2.2 ± 1.2	2.0 ± 1.0	2.4 ± 1.0
**UFFF** **Control**	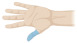			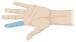			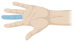			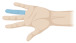		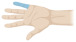			

## Data Availability

All raw data on which this study is based will be made available by the corresponding author upon request.

## References

[1] Thiem D.G.E., Frick R.W., Goetze E., Gielisch M., Al-Nawas B., Kammerer P.W. (2020). Hyperspectral analysis for perioperative perfusion monitoring-a clinical feasibility study on free and pedicled flaps. Clin. Oral Investig..

[2] Hölzle F., Wolff K.D., Mohr C. (2008). Reconstructive oral and maxillofacial surgery. Dtsch. Arztebl. Int..

[3] Lovie M.J., Duncan G.M., Glasson D.W. (1984). The ulnar artery forearm free flap. Br. J. Plast. Surg..

[4] Soutar D.S., Scheker L.R., Tanner N.S.B., McGregor I.A. (1983). The radial forearm flap: A versatile method for intra-oral reconstruction. Br. J. Plast. Surg..

[5] Loeffelbein D.J., Al-Benna S., Steinstrasser L., Satanovskij R.M., Rohleder N.H., Mucke T., Wolff K.D., Kesting M.R. (2012). Reduction of donor site morbidity of free radial forearm flaps: What level of evidence is available?. Eplasty.

[6] Bertino G., Lepenne Y., Tinelli C., Giordano L., Cacciola S., Di Santo D., Occhini A., Benazzo M., Bussi M. (2019). Radial vs ulnar forearm flap: A preliminary study of donor site morbidity. Acta Otorhinolaryngol. Ital..

[7] Heimes D., Becker P., Thiem D.G.E., Kuchen R., Kyyak S., Kammerer P.W. (2021). Is hyperspectral imaging suitable for assessing collateral circulation prior radial forearm free flap harvesting? Comparison of hyperspectral imaging and conventional allen’s test. J. Pers. Med..

[8] Sieg P., Bierwolf S. (2001). Ulnar versus radial forearm flap in head and neck reconstruction: An experimental and clinical study. Head Neck.

[9] Sieg P., Dericioglu M., Hansmann C., Jacobsen H.C., Trenkle T., Hakim S.G. (2012). Long-term functional donor site morbidity after ulnar forearm flap harvest. Head Neck.

[10] Hekner D.D., Abbink J.H., van Es R.J., Rosenberg A., Koole R., Van Cann E.M. (2013). Donor-site morbidity of the radial forearm free flap versus the ulnar forearm free flap. Plast. Reconstr. Surg..

[11] Halama D., Dreilich R., Lethaus B., Bartella A., Pausch N.C. (2019). Donor-site morbidity after harvesting of radial forearm free flaps-comparison of vacuum-assisted closure with conventional wound care: A randomized controlled trial. J. Cranio-Maxillofac Surg..

[12] Shimada K., Ojima Y., Ida Y., Komiya T., Matsumura H. (2022). Negative-pressure wound therapy for donor-site closure in radial forearm free flap: A systematic review and meta-analysis. Int. Wound J..

[13] Thiem D.G.E., Romer P., Blatt S., Al-Nawas B., Kammerer P.W. (2021). New approach to the old challenge of free flap monitoring-hyperspectral imaging outperforms clinical assessment by earlier detection of perfusion failure. J. Pers. Med..

[14] Holmer A., Marotz J., Wahl P., Dau M., Kammerer P.W. (2018). Hyperspectral imaging in perfusion and wound diagnostics—Methods and algorithms for the determination of tissue parameters. Biomed. Tech..

[15] Bickler P.E., Feiner J.R., Rollins M.D. (2013). Factors affecting the performance of 5 cerebral oximeters during hypoxia in healthy volunteers. Anesth. Analg..

[16] Gummesson C., Atroshi I., Ekdahl C. (2003). The disabilities of the arm, shoulder and hand (dash) outcome questionnaire: Longitudinal construct validity and measuring self-rated health change after surgery. BMC Musculoskelet. Disord..

[17] Franchignoni F., Vercelli S., Giordano A., Sartorio F., Bravini E., Ferriero G. (2014). Minimal clinically important difference of the disabilities of the arm, shoulder and hand outcome measure (dash) and its shortened version (quickdash). J. Orthop. Sports Phys. Ther..

[18] Tonks A.M., Lawrence J., Lovie M.J. (1995). Comparison of ulnar and radial arterial blood-flow at the wrist. J. Hand Surg. Br..

[19] Keen J.A. (1961). A study of the arterial variations in the limbs, with special reference to symmetry of vascular patterns. Am. J. Anat..

[20] Coleman S.S., Anson B.J. (1961). Arterial patterns in the hand based upon a study of 650 specimens. Surg. Gynecol. Obstet..

[21] De Vicente J.C., Espinosa C., Rua-Gonzalvez L., Rodriguez-Santamarta T., Alonso M. (2020). Hand perfusion following radial or ulnar forearm free flap harvest for oral cavity reconstruction: A prospective study. Int. J. Oral. Maxillofac. Surg..

[22] Patsalis T., Hoffmeister H.E., Seboldt H. (1997). Arterial dominance of the hand. Handchir. Mikrochir. Plast. Chir..

[23] Harding K., Queen D. (2017). Education and improved clinical outcomes. Int. Wound J..

[24] Chio E.G., Agrawal A. (2010). A randomized, prospective, controlled study of forearm donor site healing when using a vacuum dressing. Otolaryngol. Head Neck Surg..

[25] Schwarzer C., Mucke T., Wolff K.D., Loeffelbein D.J., Rau A. (2016). Donor site morbidity and flap perfusion of subfascial and suprafascial radial forearm flaps: A randomized prospective clinical comparison trial. J. Cranio-Maxillofac. Surg..

[26] Riecke B., Kohlmeier C., Assaf A.T., Wikner J., Drabik A., Catala-Lehnen P., Heiland M., Rendenbach C. (2016). Prospective biomechanical evaluation of donor site morbidity after radial forearm free flap. Br. J. Oral. Maxillofac. Surg..

[27] Yalamanchili S., Rotatori R.M., Ovalle F., Gobble R. (2020). Radial Forearm Flap Donor Site Morbidity: A Systematic Review. J. Aesthet. Reconstr. Surg..

[28] Molteni G., Gazzini L., Bisi N., Nocini R., Ferri A., Bellanti L., Marchioni D. (2022). Donor site aesthetic and functional outcomes of radial forearm free flap: A comparison between full-thickness and split-thickness skin grafts. Eur. J. Plast. Surg..

[29] Brown M.T., Couch M.E., Huchton D.M. (1999). Assessment of donor-site functional morbidity from radial forearm fasciocutaneous free flap harvest. Arch. Otolaryngol. Head Neck Surg..

[30] De Witt C.A., de Bree R., Verdonck-de Leeuw I.M., Quak J.J., Leemans C.R. (2007). Donor site morbidity of the fasciocutaneous radial forearm flap: What does the patient really bother?. Eur. Arch. Oto-Rhino-Laryngol..

